# Corrosion Resistance of Stainless Steels Intended to Come into Direct or Prolonged Contact with the Skin

**DOI:** 10.3390/ma12060987

**Published:** 2019-03-25

**Authors:** Rene Ziegenhagen, Lucien Reclaru, Lavinia Cosmina Ardelean, Alexandru Florian Grecu

**Affiliations:** 1 Richemont International SA, 3 Route des Biches, CH 1752 Villars-sur-Glânes, Switzerland; rene.ziegenhagen@richemont.com; 2 Varinor Matériaux SA, 7 St-Georges str, CH 2800 Delémont, Switzerland; lucien.reclaru@varinor.ch; 3 Department of Technology of Materials and Devices in Dental Medicine, “Victor Babes” University of Medicine and Pharmacy, 2 Eftimie Murgu sq, 300041 Timisoara, Romania; 4 MD Orthopaedics and Traumatology, University of Medicine and Pharmacy Craiova, 2 Petru Rares str, 200349 Craiova, Romania; alexandrugrecu@yahoo.com

**Keywords:** austenitic stainless steels, 316L, 904L, 317LMN, generalized corrosion, localized corrosion, galvanic couplings, Kendal tests, mixed potential

## Abstract

The biocompatibility of materials in contact with a living tissue becomes a puzzle in the overall picture of assessing the toxic effects of chemicals that come into contact with us. Allergic reactions to substances are a significant and growing health problem affecting large parts of the population in Europe. Wristwatches are objects worn in prolonged contact with the skin, being subject to localized corrosion, especially pitting and crevice types, in sulfide-chloride medium, and high wear in the bracelets joints. Watches of medium quality are usually made of stainless steels. The X2 CrNiMo 17-12-2 316L grade as well as X1 CrNiMo 20-25-5 Cu 1 or 904L are commonly used, having good resistance to generalized corrosion. The passive layer is nevertheless insufficient to ensure complete immunity in all cases of localized corrosion encountered during wear. For this reason, a high-corrosion-resistant steel: X1 CrNiMo 18-15-4 N 0.15 or 317LMN, from three different suppliers was evaluated. Metallographic characterization was carried out. The corrosion behavior evaluation was performed for the generalized corrosion, pitting and crevice corrosion and galvanic corrosion. Galvanic couples steel 317LMN-gold 18K alloy 3N and gold 18K 5M were used. The results of the generalized and pitting corrosion test indicated three basic groups. All of the 317LMNs were similar. The 316L variants tested noticeably worse. The 904Ls were difficult to discern, but certainly easier than the 316Ls and, possibly, at least comparable to the 317LMNs.

## 1. Introduction

Since 2006, Europe has a new vision regarding chemicals toxicology to humans, because of the REACH regulation (registration, evaluation, authorization, and restriction of chemicals) [1,2].

Thus the biocompatibility of materials in contact with a living tissue becomes a puzzle in the overall picture evaluating the effects of chemicals toxicity that come into contact with us [3,4,5].

ECHA (European Chemical Agency) [6] has developed a plan for the implementation of the substances of very high concern (SVHC) [7], namely endocrine disruptors (ED), carcinogens, mutagens, toxics for reproduction (CMR), and sensitizers (skin sensitizers and respiratory sensitizers). Therefore, we have to reconsider our system for assessing the toxicology of substances, mixtures of substances and devices in our homes. Among the SVHC substances incriminated by ECHA, the most frequent ones found in our homes are the skin sensitizers and respiratory sensitizers. About 4000 substances which can cause a contact allergy are listed. It is estimated that 15–20% of Europe’s population is sensitized to allergens. Allergic reactions to substances in products and devices, in both professional and private life, are a significant and growing health problem affecting large parts of the European population. Contact allergens cause dermatitis and respiratory allergens, asthma, and rhinitis [8].

Different chemicals from different sources, released at different times and from different places can expose humans to a mixture of chemicals, inducing the ‘cocktail effect’. The way chemicals are released from different sources on the one hand, and how they combine to give rise to human exposure with adverse effects on the other hand, has to be understood [9,10]. An aggravating factor is that individual chemicals can become more dangerous when mixed with other chemicals, the ‘cocktail effect’. At the moment there are no legal requirements for manufacturers to evaluate the combination of effects and risks of chemicals due to combined exposure [11]. However, the modalities of such an assessment are being reviewed by ECHA.

In this context, watch making is confronted with respecting the REACH regulations as regards consumer protection. REACH imposes the prohibition of using toxic substances such as Pb, Be, Cd, Hg, As, Se, and CrVI in consumption goods [12]. Nickel has been the subject of two European Directives, 94/27/EC and 2004/96/EC [13,14,15,16]. Today restriction conditions concerning the manufacture, marketing, and use of certain dangerous substances, mixtures and articles are found in Annex XVII REACH [17], which stipulates that these shall not be used:

(a) in any items which are inserted into pierced ears and other pierced parts of the human body, unless the rate of nickel release is less than 0.2 μg/cm^2^/week (migration limit);
(b) in articles intended to come into direct and prolonged contact with the skin such as: earrings, necklaces, bracelets and chains, anklets, finger rings, wrist-watch cases, watch straps and tighteners, rivet buttons, tighteners, rivets, zippers and metal marks, when these are used in garments, if the rate of nickel release from the parts of these articles coming into direct and prolonged contact with the skin is greater than 0.5 μg/cm^2^/week.

Our study is limited to metal materials (steel) supposed to be used in the manufacture of articles which come in prolonged contact with the skin, specific to watchmaking, such as bracelets, watchcases, etc. The steel bracelets are subjected to localized corrosion [18,19,20], in particular by pitting in a sulphide-chloride medium, and high wear in the joints of the bracelets.

Watchmaking usually uses stainless steels of the X2 CrNiMo 17-13-3 (316L) and X1 CrNiMo 20-25-5 Cu 1 (904L) grades. These stainless steels are relatively soft (typically HV 200) and have good resistance to generalized corrosion [21,22]. On the other hand, steels are sensitive to pitting and crevice corrosion, especially the 316L type [23,24]. For these reason, another alloy, X1 CrNiMo 18-15-4 N 0.15 (317LMN) was chosen as the subject of this study. This alloy has a higher hardness (HV> 200), either structurally or by thermal aging, and very low abrasion degradation. The higher molybdenum content, combined with an addition of nitrogen, provides the alloy with enhanced corrosion resistance, especially in acid-chloride-containing medium. The combination of molybdenum and nitrogen also improves the alloy’s resistance to pitting and crevice corrosion. It forms a passive layer due to the presence of nitrogen in its composition [25]. It is developed by vacuum fusion and remelted, which gives it high inclusion cleanliness and reduces the risk of pitting. This alloy is suitable for use in highly aggressive media where it may also be subject to wear. 

## 2. Materials and Methods

### 2.1. Materials

#### 2.1.1. Steel Alloys

The study is focused on seven products from three different suppliers: Supplier 1 (USA), Supplier 2 (EU), Supplier 3 (Switzerland). The standard chemical composition of 317LMN, 904L, and 316LM alloys is given in Table 1. 

The manufacturers deliver the steels with nominal compositions. In accordance with the manufacturer, the compositions of the metals may vary as in shown in Table 1.

The chemical compositions were analyzed by XRF spectrometer, carbon analysis by IR carbon analysis, and sulfur and nitrogen by IR elemental analyzer.

#### 2.1.2. Gold Base Alloys Used in Galvanic Coupling Measurements

For manufacturing jewelry, watches and other objects to wear, so called “gold jewelry” alloys are used. The amount of gold in the alloy is perfectly defined and the standard of proportions varies from one country to another [26,27]. In Europe, the title of 18K (750‰ Gold) is predominant in the jewelry market. 

In our galvanic coupling tests, we used two low gold alloys: 18K-3N: 750‰Au 125‰Ag 125‰Cu; 18K-5N: 750‰Au 50‰Ag 200‰Cu.

### 2.2. Selection of the 317LMN Stainless Steel

The austenitic stainless steels considered are characterized by very low carbon content and significant additions of nickel, chromium, and molybdenum. They have the property of developing a passive layer under anodic conditions which is formed mainly by chromium and molybdenum oxides. Molybdenum plays a decisive role in the stability of this layer. Improving corrosion resistance cannot be achieved by increasing the amount of these elements. In fact, the most highly alloyed stainless steels can develop intermetallic precipitation during heat treatments. These are at the origin of a loss of stability of the passive layer by depletion of the chromium and molybdenum elements.

Developing grades with improved corrosion resistance and metallurgic stability can be achieved by the combination of additions of nitrogen and manganese. It is thus possible to equal the super austenitic grade 904L with a much less alloyed and mechanically stronger grade, namely 317LMN.

### 2.3. Metallography

The samples were embedded in a self-curing methyl methacrylate resin, then polished with SiC paper and finally with diamond spray (6/3/1 microns). Electrolytic etching was carried out in a bath of 100 mL H_2_O, 10 mL HCl, and 5g Cr(VI)-oxide during 5 s under 0.4 V and 0.3 A. The alloys microstructures were observed using a metallographic microscope (Polyvar Met, Reichert-Jung, Vienna, Austria). The scanning electron microscopes (Sigma, Zeiss, Jena, Germany) with an Oxford X-MAX EDX Instrument for microanalysis were also used. The analyzed samples were covered with a gold flash.

### 2.4. Evaluation of the Corrosion Behavior

The corrosion behavior evaluation of the alloys was carried out based on techniques specific to the type of corrosion considered [28,29]:

- electrochemical evaluation of the generalized corrosion by the technique of the rotating electrode [30] and taking into account, for evaluation, the American Society for Testing and Materials ASTM G3-89 [31] and ASTM G59-97 [32] standards;
- pitting and crevice corrosion according to the ASTM F746-04 [33] standard;
- galvanic corrosion [34] taking into account the mixed potential theory [35].

#### 2.4.1. Evaluation of the General Corrosion

A potentiostat–galvanostat Eg&G PARSTAT 4000 with a cell of three electrodes (the reference electrode in saturated calomel (SCE) and the counter electrode in platinum), adapted for rotating electrode measurements was used. The potentiostat is equipped with a low current interface so extends the current measurement to 80 fA range(2.5 aA resolution).The tests were carried out at the ambient temperature in an artificial sweat electrolytic solution according to European Standard EN 1811-2011 [36], with the following composition: 1 ± 0.01 g·L^−1^ urea, 5 ± 0.05 g·L^−1^NaCl, 1 ± 0.01 g·L^−1^ lactic acid, and pH = 6.5 ± 0.05 via NaOH 0.1 M and deionized water (18 MΩ·cm). The samples used are cylinders (Ø = 5 mm and L = 20 mm), polished (paper P600), washed under ultrasound (TickopurR30, Weidinger Gmbh, Eichenau, Germany), and rinsed under deionized water (18 MΩ·cm) and in ethanol p.a. (Merck Co., Kenilworth, NJ, USA). The electrochemical parameters measured and calculated are:

- the open circuit potential (E_oc_) after 15 h of immersion in the electrolyte;
- the linear polarization resistance (R_p_) in domain of Mansfeld, ±20 mV SCE vs. E_oc_;
- the corrosion potential (E_corr_) and the corrosion current density (i_corr_) determined from the Tafel plots in the domain ±150 mV SCE vs. E_oc_;
- the breakdown potential (E_b_) from the potentiodynamic polarization curve between −1000 mV and +1200 mV/SCE;
- coulometric analysis by zones, the first zone between E_(I = 0)_ and +300 mV and the second zone between +300 mV and +600 mV.

#### 2.4.2. Evaluation of the Localized Corrosion

According to ASTM F746-04 [33] standard, the tests were carried out in NaCl 9 g·L^−1^ at the room temperature. The samples used were cylinders (Ø = 5 mm and L = 20 mm), polished (paper P600) which are inserted on the conical rings in polytetrafluoroethylene (PTFE). 

The test consisted first of all in an anodic excitement at +800 mV SCE for 10 s. Secondly, the potentiostat imposes a value of potential E_oc_. The potentiostatic curve is traced for 15 min. If the current registered remains in the cathodic domain, the potentiostat resumes the anodic excitement at +800 mV and will then impose a potential increase of +50 mV compared to the previous one. The cycles are repeated each time for a higher potential until the current measured remains in the anodic domain. Thus the crevice potential, E_crev_ is the potential that corresponds to the penultimate measurement where the current is positive.

#### 2.4.3. Evaluation of the Galvanic Corrosion

**Figure 1:**
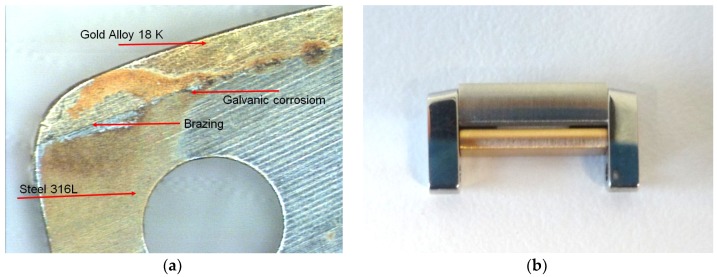
(**a**) Steel–brazing–gold 18K. (**b**) Link gold 18K-steel with pins.

Steel-gold alloy assemblies are ideal for the formation of galvanic cells [11,28]. We are dealing with significant electrical potential differences (E_couple_), which can be measured up to approximately 300 mV. A release of cations to the skin will take place. This is the typical case of nickel release, which is currently regulated by Annex XVII REACH [17]. There is also the aspect of the cathode–anode report. The surfaces of precious metals will be the cathode, and the other less noble parts will be the anode. Constructions with large cathodic surfaces and small anodic surfaces are very dangerous. The galvanic cell will discharge a strong anode current which will induce the rapid degradation of the anodic part by crevice or pitting corrosion mechanisms. This is probably the type of corrosion found most frequently in bracelets. Typical assemblies of the gold-steel links are shown in Figure 1.

There are two ways to investigate this type of galvanic corrosion: by direct measurements (E_couple_) or by prediction techniques. In the present study we used direct measurements and calculated the mixed potentials. 

##### Direct Measurements [34]

The most direct procedure involves immersing the two different metals in an electrolyte and electrically connecting them, using a zero resistance ammeter to measure the current. This technique is very accurate for time-dependent polarization, but is expensive and time consuming. In general, the couplings are measured for three to four days to obtain reliable information. Individual samples are weighed before and after the test to determine the corrosion rate as a function of potential, and thus make the necessary corrections using Faraday’s law. For this type of measurements we used the electronic assembly indicated by EG & G PAR equipped with a low current interface so extends the current measurement to 80 fA range (2.5 aA resolution). The measurements are carried out in two steps: in the first step, the open-circuit potentials of each coupling partner are recorded and in the second step the circuit is closed and the electric current discharged into the coupling is recorded.

Four samples were chosen for these measurements: #1: 317LMN** 18-14-4 MnN, #8: 316L*17-13-3 (control), #10: 904L*20-25-5 CuN(control) and #11: 904L***20-25-5 Cu.The coupling partners were low-gold alloys 18K-3N: 750‰Au 125‰Ag 125‰Cu and 750‰Au 50‰Ag 200‰Cu.

All samples were 11 mm diameter discs which are mounted in PTFE caps designed for this technique. They were obtained by spark machining (Electrical discharge machining (EDM)) technique transversely cutting from a 6 mm band. The samples are polished, washed with a mixture of acetone and ethanol, rinsed with deionized water 18 MΩ·cm and dried. 

The measurements were made in the galvanic measurement cell, the electrodes being the samples used in the direct coupling measurements. According to EN 1811-2011 [36], the electrolytic solution used is artificial sweat, at room temperature. The volume used for the electrochemical test is 600 mL.

##### Indirect Measurements

The method is based on the mixed potential theory.

**Figure 2:**
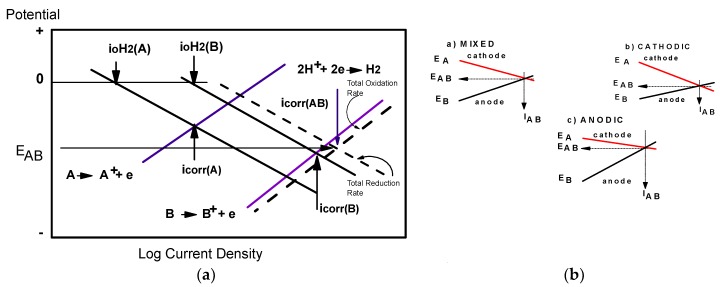
(**a**) Potential-current relationships for the case of a galvanic couple between two corroding metals. A: more noble metal; B: less noble metal [35].(**b**) Effects of polarization on metal potential (Evans diagrams).

Galvanic corrosion can be described in terms of mixed potential theory [35], as schematically illustrated in Figure 2. However, in the case of a bimetallic or multi-metal galvanic coupling in which two or more metals are electrically in contact, according to the theory there will be at least two cathodic and two anodic reactions. One of each of these reactions occurs on each metal. In this case, the most noble of the two metals will be cathodically polarized in the electronegative sense, and its anodic rate of reaction will be suppressed. Reciprocally, the less noble, or more sacrificial, more anodic material will be anodically polarized, and so the anodic rate of reaction will be accelerated. The resulting galvanic current can be determined solely from the sums of all anodic and cathodic currents for each material at each potential when the following condition is met:

i_a_·A_a_ = i_c_·A_c_ (1)

where i_a_·A_a_ is the sum of anodic currents (current density multiplied by area), and i_c_·A_c_ is the sum of the cathodic currents.

In the first step, the potentiodynamic polarization curves over a range of ±150 mV vs. SCE were drawn in the Tafel domain. In the second step, from the drawn polarization curves, a mathematical model based on the Stern–Geary equations and PAR_Calc_

χ^2^ = {[(∑ (I_obs, i_ − I_calc, i_)/s_i_]^2^}/(N − 4)}, (2)

the anodic and cathodic Tafel slopes are calculated. We retrace the rights

I(E) = I_corr_ 10 ^(E − E^_corr_^)/βa^ (3)

and

I(E) = I_corr_ 10 ^(E − E^_corr_^)/βc^ (4)

where β_a_ and β_c_ are the Tafel slopes calculated with PAR_Calc_. According to the mixed potential theory, we sum the two anodic slopes (example 3N + #8) and then the two cathodic slopes (example 3N + #8). The intersection of the anodic slope sum with the cathodic slope sum is theoretically i_coupling_ and E_coupling._

According to the Evans diagrams, the galvanic cells can work under a) mixed, b) cathodic, and c) anodic control (Figure 2b). E_oc_ and E_coupling_ are the open circuit and coupling potentials. Under these conditions, it can be seen that the E_AB_ coupling potential can approach the open-circuit potential of the E_A_ or E_B_ metal or remain somewhere between E_A_ and E_B_.

In a) we have a situation where the oxidation of the anode or the depassivation of the cathode is not really important compared to the coupling potential, thus the rates of corrosion are relatively weak. The galvanic cell is in the desirable situation of a mixed control. In b), there is a significant depassivation of the coupling partner which is in the cathode position. In c), there is a strong oxidation due to the potential difference E_B_−_AB_.

**Figure 3:**
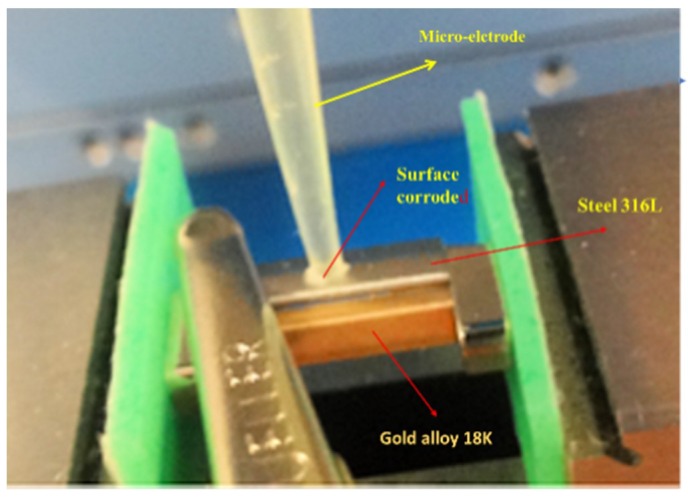
Measurement by microelectrode technique on the steel part of the bracelet.

To apply the mixed potential theory, the polarization curve (±150 mV) must be plotted for the calculation of the Tafel slopes, the corrosion potential (E_corr_) and the corrosion current (I_corr_), with a scanning speed of 0.1 mV/s. The measurements were carried out by the microelectrode technique on bracelet links (Figure 3).

### 2.5. Nickel Release—EN 1811

It is well known that Ni contact can result in an allergic response. In Europe the prevalence is 10–15% for female adults and 1–3% for male adults who are allergic to Ni. 40–70% of Ni contact- dermatitis develops acute or chronic hand eczema, estimated at 42 million people. Consequently, for the protection of the general population, in contact dermatitis, the European Union (EU) imposed the following restrictions of use:

- Ni release from parts in direct and prolonged contact with the skin must be lower than 0.5 μg/cm^2^/week [17]
- the metallic parts that are inserted into pierced ears and other parts of the human body must not have a Ni release rate greater than 0.2 μg/cm^2^/week [17].

The release of nickel cations was measured in artificial sweat according with EN 1811 (Section 2.4.1). The solutions were filtered before use over a sterilized Falcon^®^ 0.22 microns cellulose acetate membrane; the release flasks used were of Falcon^®^ sterile type made of polypropylene. The samples were in the rectangular shape of 14 mm × 35 mm and were firstly cleaned in ethanol p.a. under ultrasound. The ratio of release solution volume/total sample surface was equal to 1. The extraction was carried out at 37 °C shielded from light for 168 h. Three samples of 317LMN** 18-14-4-MnN, 317LMN* 18-15-4N (standard), 316L*** 17-13-3, 904L***20-25-5 Cu steel alloys were used. The solutions were analyzed by ICP-MS Perkin Elmer. Two blank samples were measured as a reference.

## 3. Results and Discussion

### 3.1. Metallographic Characterization

**Figure 4:**
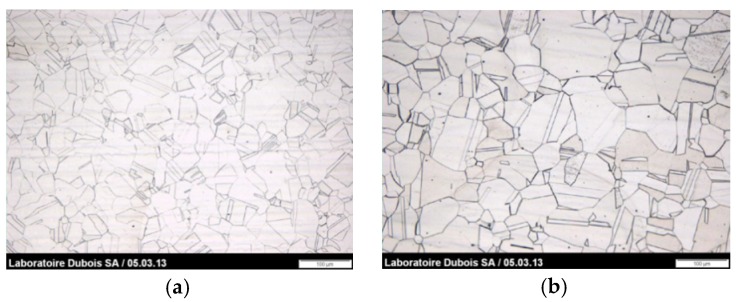

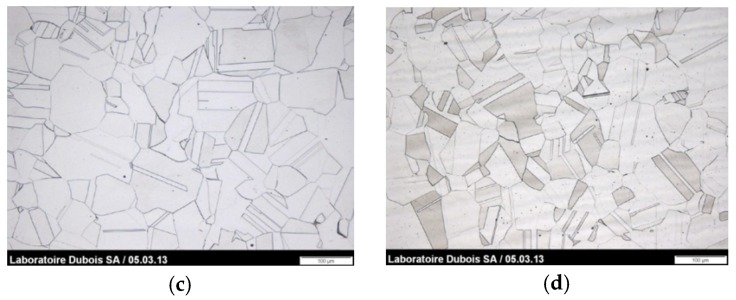
Metallographic structures, performed by optical microscopy, for # 1, # 5, # 7, and # 8 steels. (**a**) #1 317LMN: Supplier 2; (**b**) #8 316L: Supplier 1; (**c**) #5 317LMN: Supplier 1; (**d**) #7 317LMN: Supplier 1.

The metallographic structures, analyzed with optical microscopy in transverse section, are presented in Figure 4. Table 2 shows grain size and hardness values for reference steels.

The metallographic structural characteristics of the steels studied were mandated to Laboratoire Dubois, La Chaux-de-Fonds, Switzerland.

All microstructures are of recrystallized, equiaxed type. The grain size was evaluated according to ASTM E112. All steels have an average grain size of index 5. The inclusion cleanliness was evaluated according to DIN 50602 method K and the composition of the inclusions was analyzed by microprobe EDX. None of the steels has inclusions within the standard range. Inclusions of globular oxides are nevertheless present with an index less than K0 = 0. The Vickers hardness was measured by micro durometer according to ISO 6507. The measured samples show a homogeneous hardness. The average hardness ranges from 140 to 180 Vickers under load of 1 kg. This extent is to be compared to the different rates of nitrogen and molybdenum of the different grades.

### 3.2. Evaluation of the General Corrosion

Electrochemical parameters measured and calculated for the different grades of steel are given in Table 3. The breakdown potential, E_b_ is the potential for which the anodic current increases strongly.

#### 3.2.1. Evolution of the Open Circuit Potential Versus Time (E_oc_)

The open-circuit potential, E_oc_ characterizes the electrochemical state of the interface after 15 h of immersion in the absence of any polarization. The values of E_oc_ are given in Table 3.

The highest open-circuit potentials are observed for #7 and #11 steels. The following results were obtained: high positive E_oc_ values (>50 mV) for grades #7 and #11, intermediate positive E_oc_ values (0 to 50 mV) for grades #3, #8, #12, #4, #1, and #6; negative E_oc_ values (<0 mV) for grades #10, #9, #2, and #5. 

Particularities: The E_oc_ of #7 and #11 are in the cathodic zone. The E_oc_ of grade 316L (#8 and #9) are very dissimilar. The E_oc_ of grade 904L (#10, #11, and #12) are very dissimilar. The E_oc_ of grade 317L (#1, #2, #4, #5, #6, and #7) are very dissimilar.

#### 3.2.2. Resistance to Polarization (R_p_)

Resistance to polarization, R_p_, characterizes the stability of the surface in the vicinity of the potential in open circuit (E_oc_). The resistance to polarization is calculated from the polarization curves recorded in the vicinity of the potential E_oc_. The values of R_p_ are given in Table 3. The highest polarization resistance is observed for grade #6 (2162.5 kΩ/cm^2^). We note the following classification: high R_p_ values (>600 kΩ/cm^2^) for grades #6, #2, #1, #5, and #10; intermediate R_p_ values (250 to 350 kΩ/cm^2^) for grades #12 and #7; low R_p_ values (<150 kΩ/cm^2^) for grades #4, #3, #11, #9, and #8. 

Particularities: The R_p_ of the grade 316L, the #8 is the lowest. The R_p_ of the grade 316L (#8 and #9) are similar. The R_p_ of the grade 317L (#1–#7) are dissimilar. The R_p_ of the grade 904L (#10–#12) are dissimilar. 

#### 3.2.3. Tafel Plots

The calculated values of the corrosion potential, E_corr_ and the corrosion current density, i_corr_, are given in Table 3. The corrosion potential E_corr_ characterizes the zero current electrochemical state of the Tafel scanning interface. The corrosion current density, i_corr_, characterizes the corrosion intensity at Tafel’s scanning corrosion potential (i_corr_ = i_an_ − i_cat_).

Corrosion potential (E_corr_).The highest corrosion potential is observed for grade #7.We note the following classification: a high positive E_corr_ value (>50 mV) for shade #7; intermediate positive E_corr_ values (0 to 50 mV) for grades #3, #4, #8; negative E_corr_ values (<0 mV) for grades #9, #6, #10, #2, #1, #5, #11, #12. 

Particularities: The E_corr_ of the grade 904L (#12) is the lowest. The E_corr_ of the 904L grade (#11 and #12) are similar. The E_corr_ of grade 316L (#8 and #9) are dissimilar. The E_corr_ of the grade 317L (#1–#7) are dissimilar. 

Corrosion current (i_corr_).Corrosion current densities are in the range of μA/cm^2^. The lowest values are observed for samples #6, #5, and #2. We find the following classification: low i_corr_ values (<0.01 µA/cm^2^) for grades #6, #5, #2, #10, and #1; intermediate i_corr_ values (0.01 to 0.1 µA/cm^2^) for grades #3, #4, and #12; high i_corr_ values (>0.2 µA/cm^2^) for grades #11, #7, #8, and #9. 

Particularities: The i_corr_ of the grade 317L (#1–#7) are dissimilar. The i_corr_ of the grade 316L (#8 and #9) are dissimilar. The i_corr_ of the grade 904L (#10, #11, and #12) are dissimilar.

#### 3.2.4. Potentiodynamic Curves 

**Figure 5:**
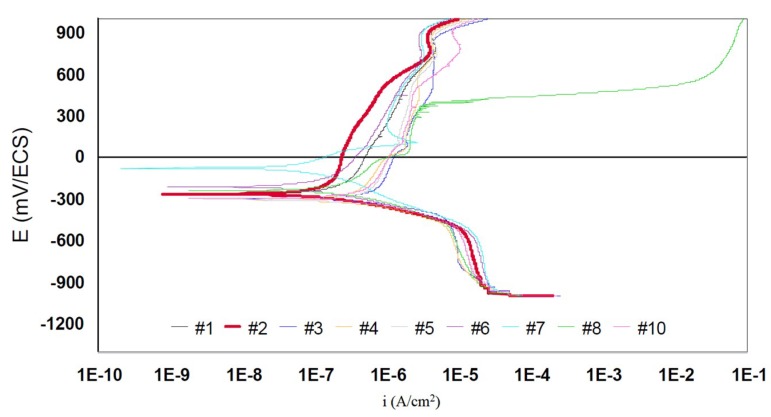
Semi-logarithmic potentiodynamic curves for all grades.

Potentiodynamic curves characterize the general electrochemical behavior of the scan interface from −1000mV to +1200mV/SCE. For the interpretation of the results two representations are used: a semi-logarithmic coordinate representation and a linear coordinate representation. Figure 5 represents the semi-logarithmic global polarization curves plotted for the samples tested in generalized corrosion. Passivation levels are noted for all samples tested for generalized corrosion. The best behavior is shown by grade #2. Intermediate behavior is revealed by grades #1, #6, and #7. Their anodic curves move to the right with currents five times higher than for alloy #2. The worst behavior is shown by grade #8. Its anode curve moves strongly to the right at currents of the order of tenth microamperes.

**Figure 6:**
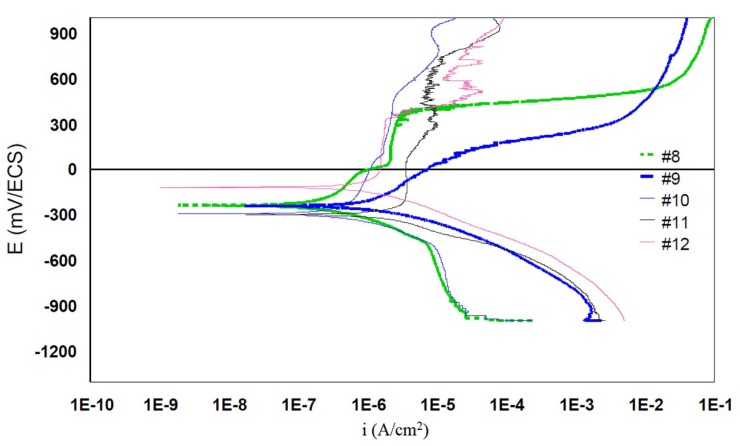
Semi-logarithmic potentiodynamic curves for the 316L (#8 and #9) grades and 904L (#10, #11, and #12) grades, which are currently used for items in contact with the skin.

The anodic currents of 316L are very high (Figure 6), which corresponds to a very fast corrosion compared to the other grades considered.

The breakdown potential E_b_ is the potential for which the anodic current increases strongly. The range of potential situated between the E_corr_ or E_(i = 0)_ and E_b_ represents the immunity zone in which the corrosion is weak, indeed insignificant. The more this zone is extended, the less the alloy is likely to be found in a situation that may lead to severe corrosion, by polarization or by galvanic effect, as a result of another alloy’s presence.

**Figure 7:**
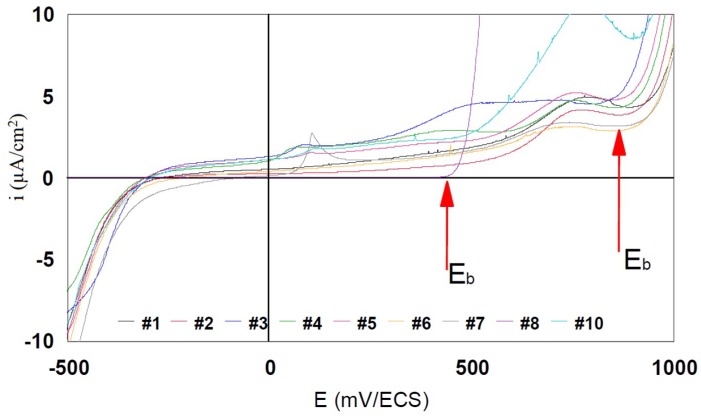
Potentiodynamic curves in linear axes for 317LMN, 904L, and 316L steels.

**Figure 8:**
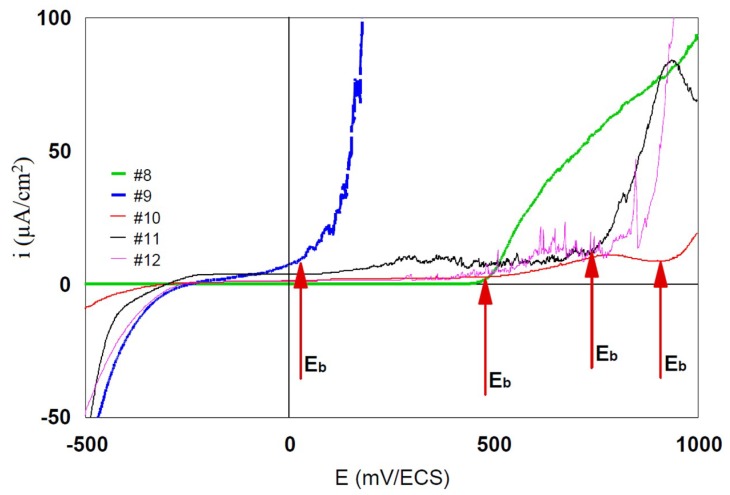
Linear potentiodynamic curves of 316L (#8 and #9) and 904L (#9, #10, and #11) steels, usually used in watchmaking.

Figure 7 and Figure 8 represent in linear coordinates the part of the polarization curve between −500 mV and +1000 mV in the cathodic–anodic current scale of between −10 μA/cm^2^ and 10 μA/cm^2^ to observe the breakdown potential (E_b_).

The E_b_ of the grade #8 is around 450 mV. The immunity domain is restricted to median potentials. Corrosion will start from 450 mV without following passivation. The E_b_ of the other grades are around 800 mV. The immunity domain reaches high potentials with low anodic currents of the order of the micro-amperes. 

E_b_ values for all studied alloys are presented in Table 3.

The results of the generalized corrosion test point out three basic groups. All of the 317LMNs were similar. The tested 316L steels were noticeably worse. The 904Ls were difficult to discern, but certainly better than the 316Ls and likely at least comparable to the 317LMNs. These conclusions are supported by data shown in Table 3. 

#### 3.2.5. Coulometric Analysis

Coulometric analysis characterizes the amount of corrosion developed in the given anodic scan range. The area of E_(i = 0)_ at +300 mV corresponds to the usual corrosion range. The area from +300 mV to +600 mV corresponds to a severe corrosion area. The results are presented in Table 3. The results are coherent between the two ranges considered. They confirm the observations and remarks made on the electrochemical magnitudes presented previously. In particular (for the second zone): the lowest electrical consumptions (<1.00 mC/cm^2^) are observed on grades #2, #6, #7, and #1; the intermediate electrical consumptions (1 to 250 mC/cm^2^) are observed on grades #5, #10, #4, #3, #12, and #11; the highest electrical consumption (>250 mC/cm^2^) is observed on grade 316L (#8 and #9). 

### 3.3. Evaluation of the Localized Corrosion

**Figure 9:**
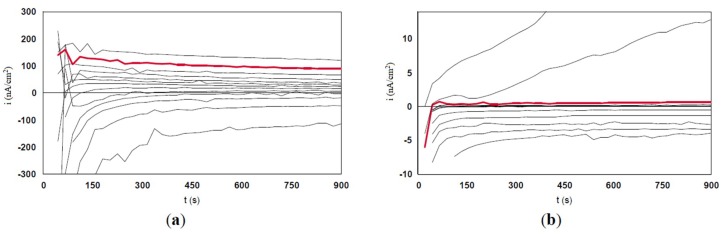
Potentiostatic scan curves for #2 and #11steels evaluated for crevice-pitting corrosion.(**a**) #2- 317LM* 20-19-4; (**b**) #11-904L*** 20-25-5 Cu.

The crevice potential, E_crev_ is the potential that corresponds to the penultimate measurement where the current is positive (potentiostatic static curve in red color). For a better understanding of the evaluation procedure of E_crev_, the potentiostatic scan curves for #2 and #11 steels are shown in Figure 9. The values for E_crev_ are summarized in Table 4.

**Figure 10:**
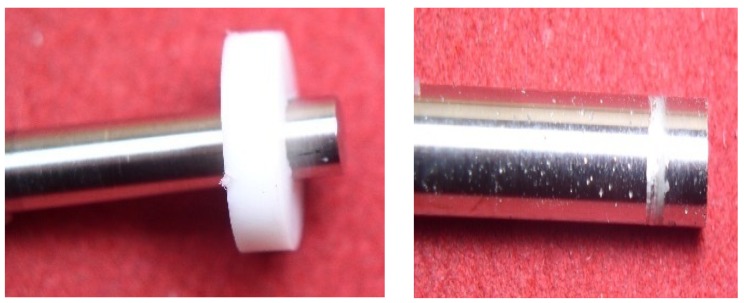
Sample #8 after the crevice-pitting corrosion test.

Figure 10 presents sample #8 (316L, control) after the crevice-pitting corrosion test. This sample was most marked by localized corrosion, its surface showing a large number of pits. The other samples do not show any pitting and the trace of crevice corrosion is not visible to the naked eye. 

These results reveal a good crevice corrosion behavior for the 317LMN alloy grades. If we classify the 317L steel grades according to their E_crev_, the best behavior is that of alloy #2, 317LM* 20-19-4. It is followed by grades #1, 317LMN** 18-14-4 MnN, #4, 317LMN* 20-18-3N and #5, 317LMN* 20-19-4N.Alloys #3, 317LM* 20-19-4 Cu (E_crev_ = 400 mV) and #6, 317LMN* 18-17-4N (E_crev_ = 350 mV) exhibit intermediate behaviors. Alloy #7, 317 LMN * 18-15-4N (standard) has the poorest behavior of the 317LMN grades (E_crev_ = 250 mV).

Steels #8, 316L* (standard) and #9, 316L*** show the poorest behavior. In general, the 316L steels are sensitive to localized corrosion by pitting and crevices. Thus the quantities of nickel released are higher than the 0.5 micrograms/cm^2^ imposed by the standards of protection concerning nickel allergies.

Alloy #10, 904L* (control) has a crevice potential of 350 mV, higher than the crevice potential of sample #11, 904L*** 20-25-5 Cu. Alloy #12, 904L*** 20-25-5 Cu exhibits a similar behavior (E_crev_ = 350 mV) to #11. This steel grade begins to replace the type 316L grades in the high watchmaking range. They have good corrosion resistance and the quantities of nickel released are in compliance with the current legislation in the EU, USA, Australia, Canada, Asia, etc.

### 3.4. Non-Parametric Kendal Test 

We measured and evaluated six electrochemical parameters: E_oc_, R_p_, E_corr_, i_corr_, and coulometric analyses for both zones. Since the experimental conditions are strictly the same for all the alloys tested, a global view of the corrosion behavior can be given by a non-parametric classification of the Kendall rank correlation type [37,38,39]. Table 5 is created in the following way: each quantity is ordered by increasing value. Each of the 12 values is assigned a rank from 1 to 12. Rank 1 represents the best behavior, rank 12 represents the worst behavior. The resulting rank is then assigned to the corresponding steel (#1–#12). Once the Table 5 is filled, we calculate the sum of the ranks for each grade (Σ_rang_). Theoretically, the highest performance would correspond to the index Σ = 6, (6 parameters x rank 1), and the lowest performance to the index Σ = 72, (6 parameters x rank 12). Without calculating the Kendall coefficients, we notice that we can form four classes.

The classification of grades after the generalized corrosion results allows the following observations: the best behaviors are shown by the 317LMN grades; the best 317LMN grades, #6, #2, and #7 are close to the ‘standard’ 18% chromium composition; grades 317LM and 317LMN‘alloyed’ with 20% chromium, #1, #4, #3, and #5 are in the intermediate position, together with the grade 904L #10; the grades 904L #11, #12 and the grades 316L #8 and #9 belong to the last classes. In the same way, we can classify the steels according to the results of crevice corrosion tests. The classification parameter is the crevice corrosion potential (E_crev_). In Table 6, all the values of an equal series carry the same rank as the smallest index and the higher index ranks of this series are deleted. In addition, crevice corrosion is considered to be twice as serious as generalized corrosion on the products under consideration. The ranks of E_crev_ are thus affected by a factor of 2. 

The ordering of the grades after crevice corrosion results leads to the following observations: the best behaviors are shown by the 317LMN grades; the best 317LMN grades, #2, #4, #5, #3, are the 317LM and 317LMN grades, ‘alloyed’ with 20% chromium; the grades 317LMN #6, #1, #7, of ‘standard’ composition with 18% chromium, are in intermediate position with the grades 904L #10 and #11; grades 316L #8 and #9 are in last position. Note the order inversion of the 317LMN ‘standard’ and 317LMN ‘alloyed’ grade groups between generalized corrosion and crevice corrosion. 

To obtain an overall classification of generalized corrosion and crevice corrosion, the rank values obtained in crevice corrosion after multiplication can be added to the rank sum of Table 7.

Table 7 shows rank changes related to the influence of crevice corrosion: the best behavior is shown by 317LM #2 followed by 317LMN #6 and #1 (class I); steels 317LMN #4, #5, # 7, #3, and 904L #10 (control) are found in Class II; 904L steel and 316L steel keep their position in Class III; lower than the grades 316L Class IV.

The comparison of the 317LMN #1 and #7 grades, however similar in composition, shows a difference, more particularly in crevice corrosion. This result can be attributed to the presence of secondary phase inclusions (sigma) in grade #7 and to the ultrapure nature of the remelted grade #1. The role of manganese in grade #1 is uncertain. The difference between grades 317LM #2 and #3 shows the adverse effect of copper in crevice corrosion.

Two hypotheses make it possible to explain the fact that the grades 904L remain lower than the grades 317LMN: on one hand the proportion of the passivating elements in the 317LMN grades can be more favorable to the formation of a stable oxide layer than in the grade 904L (kinetic effect) and on the other hand, the 904L grade may have a lack of passivation in a diluted low-oxidizing salt electrolyte, such as that used for the test. These grades remain partially active at ‘low potential’.

The difference between the grades 904L #10, #11, and #12 may be relative to a different elaboration method. A higher impurity level in grades #11 and #12 is suspected. In particular, shade #11 is developed to facilitate shavings removal.

Grade 316L #8 and #9 do not resist to crevice corrosion, as empirical observations on the products show. The proportion of passivating elements in these grades is too low to allow repassivation in crevice corrosion. These grades remain active in this corrosion configuration and have a higher probability of galvanic corrosion and nickel release.

### 3.5. Galvanic Coupling Measurements

**Figure 11:**
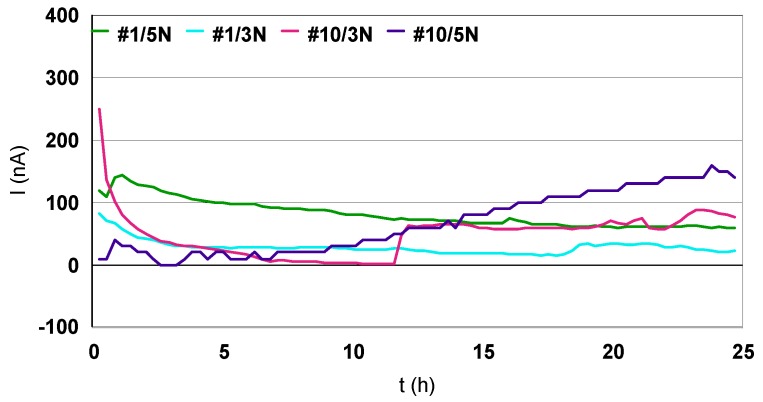
Galvanic currents measured in coupling #1/(3N, 5N)18K gold and #10/(3N, 5N)18K gold.

After 12 h of immersion, the potentials of each coupling partner were collected. No wonder the open circuit potential (E_oc_) values are specific for 18K gold alloys and steels. After gold–steel partner couplings, the galvanic current values were recorded for 25 h (Figure 11). For this relatively short period we can notice a trend, but to have more information it would be necessary to extend the measurements to 3–5 days. In this method, the galvanic current can be directly determined as a function of time. A reference electrode can be used in the usual manner to determine the galvanic couple potential.

It is obvious that the galvanic currents are in the nano-amperes scale, the galvanic currents in the #10/Au couplings are larger than in the #1/Au couplings; #10/Au galvanic currents have a tendency to rise with time; the galvanic currents #1/Au, on the contrary, tend to decrease with time; the galvanic currents generated in the Au 5N coupling are greater than in the Au 3N coupling. This is an aspect that should be the subject of further study; to establish the influence of the elements in the composition of Au 18K and the kinetics of cations release with respect to the chemical composition. 

The galvanic currents recorded can be positive or negative. This is a question of positioning the electrodes in the electronic measurement setup. It is very important to keep the same position in the assembly, the 18K gold alloy will be the cathode and the steel will be the anode. In the coupling, according to the electrochemical laws, the anode corrodes the weakest. Suppose that in this case the generated coupling current has a positive sign. If a negative current is recorded then the anode will be the 18K alloy and the cathode will be the steel. In other words the steel, passivated, becomes a cathode and corrodes Au 18K.

**Figure 12:**
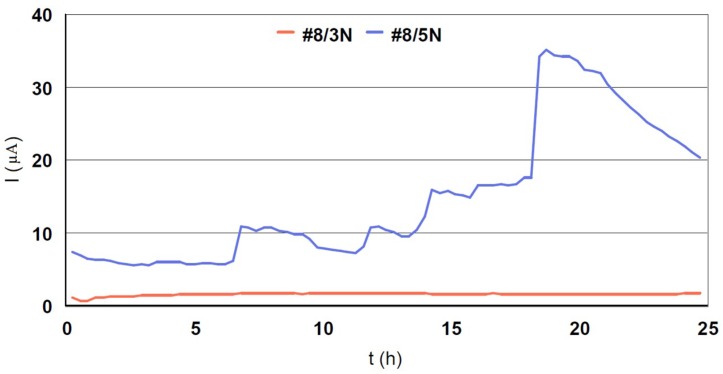
Galvanic currents measured in couplings #8/(3N, 5N)18K gold.

The galvanic currents of the #8/Au couplings are presented in Figure 12. Their order of magnitude is micro-ampers; so we built a graph in the micro-ampers scale. In the construction of an Au-steel watch bracelet, the use of a 316L type steel is not the best solution. The coupling currents generated are of the order of micro-ampers; therefore, the risk of triggering corrosion in the bracelet powered by a galvanic battery is very high. The quantities of nickel released in contact with the skin will also have to be taken into consideration [17].

#### Evaluation of Galvanic Couplings from the Mixed Potential Theory

We have plotted the potentiodynamic polarization curves in the domain of Tafel E_corrr_ ± 150 mV for gold alloys 3N, 5N, steels #1: 317LMN** 18-14-4 MnN, #8:904L* 20-25-5 CuN (control), #10: 904L* 20-25-5 CuN (control) and #11: 904L*** 20-25-5 Cu. The curves of Tafel and i_corr_ were calculated with PAR_Calc_ routine EG&G PARC Application Model SOFT Corr TMII. The Scan rate was 0.1 mV/s. The calculated values of E_corr_*,* i_corr_ in the Tafel domain are presented in Table 8. 

**Figure 13:**
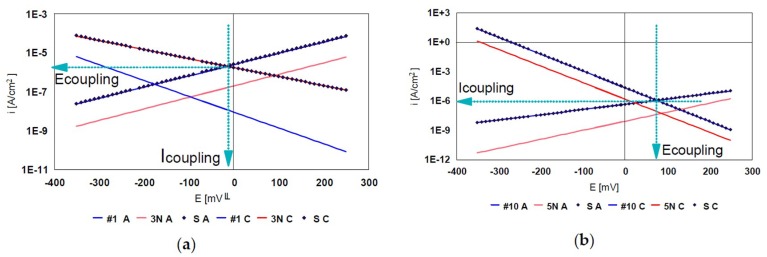
Determination of I_coupling_ and E_coupling_ for the coupling gold 18K/steels. (**a**) Coupling #1/3N; (**b**) Coupling #10/5N.

The values in Table 8 were used in the determination of E_corr_ coupling and i_corr_ coupling according to the theory of mixed potential. Figure 13 shows two coupling diagrams for steels 316L, 317LMN 3N and 5N.

**Figure 14:**
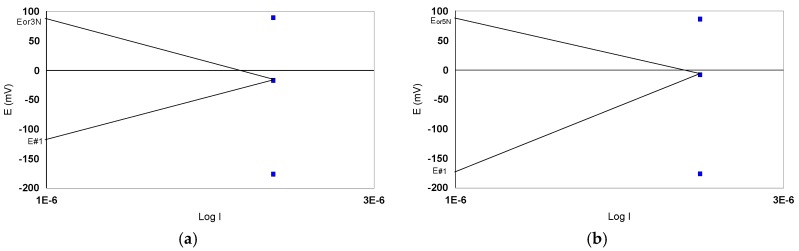
Evans diagrams drawn for studied steel/gold couplings. (**a**) Coupling #1/3N; (**b**) Coupling #1/5N.

In Table 9 the coupling values I (I_coupling_) and E (E_coupling_) calculated for each coupling are presented. It should be noted that the 316L/3N, 5N couplings confirm the current quantities measured directly and, on the other hand, the coupling values in 316L/Au 5N are larger than in 316L/Au 3N. The values in Table 9 are used to draw Evans diagrams. Thus we will be able to examine the control under which the galvanic battery functions (Figure 14).

Examination of the Evans diagrams of the couplings studied does not reveal any anodic or cathodic batteries. We can say that they are under mixed control. On the other hand, E_coupling_-E_oc_ differences are quite important. Taking into account that the measured coupling current values are of the order of 10^−6^ A, they are low. The exception is the case # 8/Au for which the currents are of the order 10^−4^–10^−5^ A.

### 3.6. Ni Release

The results for the extraction tests according to European Legislation EN 1811 are presented in Table 10.

As a result, the grades of steels studied are suitable for manufacturing items which are in prolonged contact with the skin, acceptance limit 0.5 μg/cm^2^/week. On the contrary, they are prohibited to be used to manufacture metallic parts that are inserted into pierced ears and other parts of the human body, acceptance limit 0.2 μg/cm^2^/week.

## 4. Conclusions

The corrosion behavior of all the 317LMNs was similar. All 317LMNs have proven better in general corrosion as well as in crevice and pitting localized corrosion. The 316L variants, least alloyed, behaved noticeably worse and occupy the last classes. The 904Ls were difficult to discern, but were certainly better than the 316Ls and at least comparable to the 317LMNs. 317LMN(standard) grades with 18% chromium, #6, #7, #1 are better in generalized corrosion while 317LMN grades ‘alloyed’ with 20% chromium, #2, #4, #3, and #5 proved better in crevice corrosion.

The best 317LMN grades are those alloyed with 20% chrome, #2, #4, and #5. They are better than grade #10 904L (control). The ‘standard’ 317LMN (standard) with 18% chromium, #6, #7 occupy an intermediate position between grades #10 and #11. 

The comparison of the #1 and #7 317LMN grades, however similar in composition, shows a difference, more particularly in crevice corrosion.

The 317LMN grades are better compared to the 904L grades under the corrosion conditions applied. It is reasonable to assume a negligible occurrence of Au-steel galvanic corrosion and crevice corrosion. In the case of 317LMN steels, the galvanic currents have a tendency to decrease with time, on the other hand, for 904L steel the tendency is to increase with time. There is a difference in steel/Au 3N and steel/Au 5N couplings, coupling currents with 5N are larger than those with 3N.

At present, metal watchmaking items in contact with the skin are manufactured using 316L and 904L steels. Unfortunately, when dealing with gold–steel bimetallic constructions, it is very difficult to respect the quantities of nickel released, according to international regulations. This is the reason why this research was oriented towards the development of new grades of steel that meet the new current legislation, for consumer protection.

## Figures and Tables

**Table 1:** Compositions in % weight of the alloys tested.

Code	No.	C	Cr	Ni	Mo	N	Designation EN	Comparative Designation
#1	00	0.02	17.47	13.73	4.09	0.2	X1 CrNiMo 18-14-4 Mn1 N 0.15	317LMN** 18-14-4 MnN
#2	04	0.001	19.82	18.95	4.02	0.029	X1 CrNiMo 20-19-4	317LM* 20-19-4
#3	05	0.001	19.86	19.01	4.02	0.029	X1 CrNiMo 20-19-4 Cu	317LM* 20-19-4 Cu
#4	44	0.001	20.04	18.13	3.5	0.14	X1 CrNiMo 20-18-3 N 0.15	317LMN*20-18-3 N
#5	45	0.003	20.12	19.15	4.04	0.13	X1 CrNiMo 20-19-4 N 0.15	317LMN* 20-19-4 N
#6	46	0.004	18.56	17.61	4.03	0.13	X1 CrNiMo 18-17-4 N 0.15	317 LMN* 18-17-4 N
#7	47	0.001	18.6	15.66	4.53	0.15	X1 CrNiMo 18-15-4 N 0.15	317LMN* 18-15-4 N (standard)
#8	06	0.001	16.92	12.07	2.53	0.026	X2 CrNiMo 17-12-2	316L* 17-13-3 (control)
#9	61	0.017	17.80	13.10	2.67	-	X2 CrNiMo 17-13-3	316L*** 17-13-3
#10	07	0.001	20.66	25.37	4.47	0.03	X1 CrNiMo 20-25-5 Cu1 N 0.15	904L* 20-25-5 CuN (control)
#11	71	0.02	20.22	25.60	5.20	-	X1 CrNiMo 20-25-5 Cu1	904L*** 20-25-5 Cu
#12	72	0.01	19.8	24.10	4.62	0.04	X1 CrNiMo 20-25-5 Cu1	904L*** 20-25-5 Cu

* Supplier 1 (USA); ** Supplier 2 (EU), *** Supplier 3 (Switzerland).

**Table 2:** Metallographic data.

Code	No.	Comparative Designation	Grain Size ASTM E112	Hardness ISO 6507
#1	00	317LMN** 18-14-4 MnN	6	HV1 180
#5	45	317LMN* 20-19-4 N	4.5	HV1 165
#7	47	317LMN* 18-15-4 N (standard)	5	HV1 180
#8	06	316L* 17-13-3 (control)	5	HV1 140

* Supplier 1 (USA); ** Supplier 2 (EU).

**Table 3:** Electrochemical parameters measured and calculated for the different grades of steel

Code	E_oc_ (mV)	E_corr_ (mV)	R_p_ (kΩ/cm²)	i_corr_ (µA/cm^2^)	Coulometric Analysis E_(I=0)_ − 300 mV (mC/cm^2^)	Coulometric Analysis 300–600 mV (mC/cm^2^)	E_b_ (mV)
#1	11	−94.4	785.4	0.0094	0.661	1.036	900
#2	−42	−82.4	805.9	0.0060	0.299	0.513	900
#3	32	22.95	106	0.0120	1.729	2.337	900
#4	27	15.6	126.5	0.0660	1.509	1.655	900
#5	−73	−98.7	721.9	0.0059	1.365	1.359	900
#6	6	−39.6	2162.5	0.0011	0.480	0.857	900
#7	86	79.2	259.35	0.2501	0.647	0.936	850
#8	31	12.3	14.86	0.2563	1.552	3780	450
#9	−40	−32	27	2.20	260.7	6179	100
#10	−36	−68	607.55	0.0084	1.441	1.671	350
#11	64	−105	81	0.21	5.323	4.963	700
#12	29	−118	347	0.091	2.50	20.70	700

**Table 4:** Values for the crevice potential E_crev,_ for steels tested according to ASTM F746-87.

Code	Alloy	E_crev_ (mV)
#1	317LMN** 18-14-4-MnN	450
#2	317LM* 20-19-4	500
#3	317LM* 20-19-4 Cu	400
#4	317LMN* 20-18-3N	450
#5	317LMN* 20-19-4N	450
#6	317LMN* 18-17-4N	350
#7	317LMN* 18-15-4N (standard)	250
#8	316L* 17-13-3 (control)	100
#9	316L*** 17-13-3	50
#10	904L* 20-25-5 CuN(control)	350
#11	904L*** 20-25-5 Cu	200
#12	904L*** 20-25-5 Cu	350

* Supplier 1 (USA); ** Supplier 2 (EU), *** Supplier 3 (Switzerland).

**Table 5:** Ranks attributed to 12 steels according to 6 parameters of generalized corrosion

Code	E_oc_	E_corr_	R_p_	i_corr_	I*	II**	Σ_rang_
#1	7	9	3	5	3	4	31
#2	11	8	2	3	1	1	26
#3	3	2	9	6	9	8	37
#4	6	3	8	7	7	6	37
#5	12	10	4	2	5	5	38
#6	8	6	1	1	2	2	20
#7	1	1	7	10	4	3	26
#8	4	4	12	11	8	11	50
#9	10	5	11	12	12	12	62
#10	9	7	5	4	6	7	38
#11	2	11	10	9	11	9	52
#12	5	12	6	8	10	10	51

I* Coulometric analysis E_(i = 0)_ 300 mV; II** Coulometric analysis 300–600 mV.

**Table 6:** Ranks attributed to the 12 steels according to the crevice corrosion parameter.

Code	E_crev_	Σ_rang_
#1	2	4
#2	1	2
#3	5	10
#4	2	4
#5	2	4
#6	7	14
#7	9	18
#8	12	24
#9	11	22
#10	5	10
#11	10	20
#12	7	14

**Table 7:** Kendal classification of steels in generalized corrosion and crevice corrosion.

Code	Alloy	Designation AISI Standard	Σ rang	Class
#2	X1 CrNiMo 20-19-4	317LM* 20-19-4	28	Class I
#6	X1 CrNiMo 18-17-4 N 0.15	317LMN* 18-17-4 N	34	Class I
#1	X1 CrNiMo 18-14-4 Mn1 N 0.15	317LMN**18-14-4 MnN	35	Class I
#4	X1 CrNiMo 20-18-3 N 0.15	317LMN* 20-18-3 N	41	Class II
#5	X1 CrNiMo 20-19-4 N 0.15	317LMN* 20-19-4 N	42	Class II
#7	X1 CrNiMo 18-15-4 N 0.15	317LMN* 18-15-4 N (standard)	44	Class II
#3	X1 CrNiMo 20-19-4 Cu	317LM* 20-19-4 Cu	47	Class II
#10	X1 CrNiMo 20-25-5 Cu 1 N 0.15	904L* 20-25-5 CuN (control)	48	Class II
#12	X1 CrNiMo 20-25-5 Cu 1	904L*** 20-25-5 Cu	65	Class III
#11	X1 CrNiMo 20-25-5 Cu 1	904L*** 20-25-5 Cu	72	Class IV
#8	X2 CrNiMo 17-12-2	316L* 17-13-3 (control)	74	Class IV
#9	X2 CrNiMo 17-13-3	316L*** 17-13-3	84	Class IV

* Supplier 1 (USA); ** Supplier 2 (EU), *** Supplier 3 (Switzerland).

**Table 8:** Electrochemical parameters calculated for the different grades of steel and gold alloys in the Tafel domain.

Code	Designation AISI Standard	E_corr_ (mV)	b_c_ (mV/dec)	b_a_ (mV/dec)	i_corr_ (A/cm^2^)
3N	750‰Au 125 ‰Ag 125‰Cu	90	217	169	6.8 × 10^−7^
5N	750‰Au 50 ‰Ag 200‰Cu	87	59	108	5.4 × 10^−8^
#1	317LMN** 18-14-4 MnN	−175	123	173	2.4 × 10^−7^
#8	904L* 20-25-5 CuN (control)	95	58	186	1.8 × 10^−6^
#10	316L* 17-13-3 (control)	−335	146	123	3.3 × 10^−6^
#11	904L*** 20-25-5 Cu	−126	176	484	4.7 × 10^−6^

b_c:_ cathodic Tafel slope; b_a:_ anodic Tafel slope; * Supplier 1 (USA); ** Supplier 2 (EU), *** Supplier 3 (Switzerland).

**Table 9:** Values I_coupling_ and E_coupling_ calculated according to the mixed potential theory.

Coupling	E_coupling_ (mV)	I_coupling_ (A)
#1/3N	−16	2.13 × 10^−6^
#8/3N	−234	2.18 × 10^−5^
#10/3N	−94	2.20 × 10^−6^
#11/3N	−72	6.31 × 10^−6^
#1/5N	−7	2.25 × 10^−6^
#8/5N	−119	1.86 × 10^−4^
#10/5N	75	1.24 × 10^−6^
#11/5N	−3	8.62 × 10^−6^

**Table 10:** Results of ICP analysis of the artificial sweat. Average of three measurements, (μg/cm^2^/week).

Code	Alloy	(μg/cm^2^/week)
#1	317LMN** 18-14-4-MnN	0.28
#7	317LMN* 18-15-4N (standard)	0.25
#9	316L*** 17-13-3	0.28
#11	904L***20-25-5 Cu	0.18

* Supplier 1 (USA); ** Supplier 2 (EU), *** Supplier 3 (Switzerland).

## References

[1] Regulation (EC) No 1907/2006 of the European Parliament and of the Council of 18 December 2006 concerning the Registration, Evaluation, Authorisation and Restriction of Chemicals (REACH), Establishing a European Chemicals Agency, Amending Directive 1999/45/EC and Repealing Council Regulation (EEC) No 793/93 and Commission Regulation (EC) No 1488/94 as Well as Council Directive 76/769/EEC and Commission Directives 91/155/EEC, 93/67/EEC, 93/105/EC and 2000/21/EC. https://echa.europa.eu/regulations/reach/legislation.

[2] Graedel T.E., Harper E.M., Nassar N.T., Nuss P., Reck B.K. (2015). Criticality of metals and metalloids. Proc. Natl. Acad. Sci. USA.

[3] Jaishankar M., Tseten T., Anbalagan N., Blessy B.M., Beeregowda K.M. (2014). Toxicity, mechanism and health effects of some heavy metals. Interdiscip. Toxicol..

[4] Hamann D., Hamann C.R., Thyssen J.P. (2013). The impact of common metal allergens in daily devices. Curr. Opin. Allergy Clin. Immunol..

[5] Reclaru L., Ardelean L., Rusu L.C. (2008). Toxic materials, allergens and mutagens and their impact on the dental field. Med. Evol..

[6] ECHA (European Chemical Agency). https://echa.europa.eu/home.

[7] Substances of Very High Concern Identification. https://echa.europa.eu/substances-of-very-high-concern-identification.

[8] Diepgen T.L., Ofenloch R.F., Bruze M., Bertuccio P., Cazzaniga S., Coenraads P.J., Elsner P., Goncalo M., Svensson Å., Naldi L. (2016). Prevalence of contact allergy in the general population in different European regions. Br. J. Derm..

[9] Godrey A., Abdel-Moneim A., Sepúlveda M.S. (2017). Acutemixturetoxicity of halogenated chemicals and their next generation counterparts on zebrafish embryos. Chemosphere.

[10] Meyer O. (2003). Testing and assessment strategies, including alternative and new approaches. Toxicol. Lett..

[11] Polychronis G., Al Jabbari Y.S., Eliades T., Zinelis S. (2018). Galvanic coupling of steel and gold alloy lingual brackets with orthodontic wires: Is corrosion a concern?. Angle Orthod..

[12] ECHA Substances Restricted under REACH. https://www.echa.europa.eu/web/guest/substances-restricted-under-reach.

[13] (1999). Commission Communication 20.7.1999 in the Framework of the implementation of Parliament and Council Directive 94/27/EC of 30 June 1994. Off. J. Eur. Communities.

[14] (2004). Commission Directive 2004/96/EC of 27 September 2004 amending Council Directive 76/769/EEC as regards restrictions on the marketing and use of nickel for piercing post assemblies. Off. J. Eur. Union.

[15] (2016). Commission Communication in the framework of the implementation of Regulation (EC) No 1907/2006 of the European Parliament and of the Council concerning the Registration, Evaluation, Authorisation and Restriction of Chemicals (REACH). Off. J. Eur. Union.

[16] (2007). Commission communication as regards restrictions on the marketing and use of nickel. Off. J. Eur. Union.

[17] Annex XVII REACH. https://echa.europa.eu/documents/10162/7851171d-53e9–455a-8bb8–7ca22e89ad87.

[18] ISO 23160:2011 Watch Cases and Accessories—Tests of the Resistance to Wear, Scratching and Impacts. https://www.iso.org/standard/45522.html.

[19] Haudrechy P., Mantout B., Frappaz A., Rousseau D., Chabeau G., Faure M., Claudy A. (1997). Nickel release from stainless steels. Contact Dermatitis.

[20] Reclaru L., Ziegenhagen R., Eschler P.Y., Blatter A., Lemaître J. (2006). Comparative corrosion study of “Ni-free” austenitic stainless steels in view of medical applications. Acta Biomater..

[21] Ha H.Y., Jang J.H., Lee T.H., Won C., Lee C.H., Moon J., Lee C.G. (2018). Investigation of the localized corrosion and passive behavior of type 304 stainless steels with 0.2–1.8 wt % B. Materials.

[22] Reclaru L., Lerf R., Eschler P.Y., Blatter A., Meyer J.M. (2002). Pitting, crevice and galvanic corrosion of REX stainless-steel/CoCr orthopedic implant material. Biomaterials.

[23] Ju H., Duan J., Yang Y., Cao N., Li Y. (2018). Mapping the Galvanic Corrosion of Three Coupled Metal Alloys Using Coupled Multielectrode Array: Influence of chloride ion concentration. Materials.

[24] Huang Y., Wu W., Cong S., Ran G., Cen D., Li N. (2018). Stress corrosion behaviors of 316LN stainless steel in a simulated PWR primary water environment. Materials.

[25] Alloy 317LMN Austentitic Stainless Steel Plate-Sandmeyer Steel. https://www.sandmeyersteel.com/317LMN.html.

[26] ISO 8654: 2018 Jewellery—Colors of Gold Alloys—Definition, Range of Colors and Designation. https://www.iso.org/standard/65409.html.

[27] 941.311 Ordonnance sur le contrôle du commerce des métauxprécieux et des ouvragesenmétauxprécieux (Ordonnance sur le contrôle des métauxprécieux, OCMP) du 8 mai 1934 (Etat le 2 août 2013) Suisse. https://www.admin.ch/ch/f/rs/c941_311.html.

[28] Baboian R., France W., Rowe L., Rynewicz. J. (1976). Galvanic and Pitting Corrosion—Field and Laboratory Studies.

[29] Landolt D. (1993). Corrosion et Chimie de Surfaces des Métaux (Traité des Matériaux).

[30] Reclaru L., Ziegenhagen R., Unger R.E., Eschler P.Y., Constantin F. (2014). New generation super alloy candidates for medical applications: Corrosion behavior, cation release and biological evaluation. Mater. Sci. Eng..

[31] ASTM G3-89 (2004). Standard Practice for Conventions Applicable to Electrochemical Measurements in Corrosion Testing. https://www.astm.org/Standards/G89.htm.

[32] ASTM G59-97 (2014). Standard Test Method for Conducting Potentiodynamic Polarization Resistance Measurement. https://www.astm.org/Standards/G59.htm.

[33] ASTM 746-04 (2014). Standard Test Method for Pitting or Crevice Corrosion of Metallic Surgical Implant Materials. https://www.astm.org/Standards/F746.htm.

[34] ASTM G71—81 (2014). Standard Guide for Conducting and Evaluating Galvanic Corrosion Tests in Electrolytes. https://www.astm.org/Standards/G71.htm.

[35] ASTM G82-98 Standard Guide for Development and Use of a Galvanic Series for Predicting Galvanic Corrosion Performance. https://www.astm.org/Standards/G82.htm.

[36] (2011). EN 1811-2011, Reference Test Method for Release of Nickel from All Post Assemblies which are Inserted Into Pierced Parts of the Human Body and Articles Intended to Come Into Direct or Prolonged Contact With the Skin, CEN/TC347. https://www.en-standard.eu/din-en-1811.

[37] Abdi H. The Kendall rank correlation coefficient. https://www.utdallas.edu/~herve/Abdi-KendallCorrelation2007-pretty.pdf.

[38] Kendall M.G. (1955). Rank Correlation Methods.

[39] Siegel S. (1956). Nonparametric Statistics for the Behavioral Sciences.

